# Effects of Integrating Jaw Opening and Closing Movements with Active Neck Exercises in the Management of Chronic Non-Specific Neck Pain: A Randomized Controlled Trial

**DOI:** 10.3390/medicina60091437

**Published:** 2024-09-03

**Authors:** Saeed Akhter, Hamayun Zafar, Ashfaq Ahmad, Waqas Ahmed Farooqui

**Affiliations:** 1 Department of Physiotherapy, Sindh Institute of Physical Medicine & Rehabilitation, Chand Bibi Road, Karachi 74200, Pakistan; 2 University Institute of Physical Therapy, The University of Lahore, Lahore 54400, Pakistan; hzafar@ksu.edu.sa (H.Z.); ashfaq.ahmad@uipt.uol.edu.pk (A.A.); 3 Department of Rehabilitation Sciences, College of Applied Medical Sciences, King Saud University, Riyadh 11433, Saudi Arabia; 4 Department of Odontology, Clinical Oral Physiology, Faculty of Medicine, Umea University, 901 87 Umea, Sweden; 5 School of Public Health, Dow University of Health Sciences, Karachi 74200, Pakistan; waqas.ahmed@duhs.edu.pk

**Keywords:** neck pain, temporomandibular joint, intervention, exercise, physiotherapy

## Abstract

*Background and Objectives*: It has been seen that jaw opening is associated with neck extension and jaw closing is associated with neck flexion. This natural association between the jaw and neck can be used as a novel approach to treat chronic non-specific neck pain, although the effects of this concept have never been previously evaluated as a treatment strategy. This article intends to study the effects of integrating jaw opening and closing movements along with active neck exercises versus active neck exercises alone in the management of chronic non-specific neck pain. *Materials and Methods*: A total of 80 patients, aged 20 to 50, with chronic non-specific neck pain were included in a double-blind randomized controlled trial, conducted at the Sindh Institute of Physical Medicine and Rehabilitation, Karachi, Pakistan from 2018 to 2022. The patients were divided into two groups: Group A patients were assigned jaw movements with active neck exercises, while Group B patients were assigned only active neck exercises. Both groups were assigned isometric strengthening exercises and self-resisted strengthening exercises for cervical spine muscles as a home plan. The study used various outcome measures, including the numerical pain rating scale (NPRS), neck disability index (NDI), neck flexion endurance (NFE), neck extension endurance (NEE), the neck proprioception error (NPE): neck flexion proprioception error (NFPE), neck extension proprioception error (NEPE), neck right rotation proprioception error (NRRPE), and neck left rotation proprioception error (NLRPE), with measurements taken at week 1 and week 6, respectively; the mean differences between the groups were measured using a two-way repeated ANOVA. *Results*: The experimental group showed better improvements compared to the control group, NPRS (73%), NDI (57%), NFE (152%), NEE (83%), NFPE (58%), NEPE (65%), NRRPE (65%), and NLRPE (62%), with a significant difference (*p* < 0.05). *Conclusions*: Active neck extension and flexion movements combined with jaw opening and closing are more effective in reducing pain and disability, improving neck muscles endurance and normalizing neck proprioception in patients with chronic neck pain.

## 1. Introduction

Neck pain is one of the most common musculoskeletal conditions, which affect 16.7% to 75.1% of the general population worldwide each year. In India, the figure is about 45.2%, and in Pakistan, 16.8% has been reported [1,2,3]. One of the most prevalent of these musculoskeletal conditions is neck pain, which had an age-standardized prevalence rate of 27.0 per 1000 people in 2019 [4]. The diagnosis of non-specific neck pain (NNP) is purely based on clinical examination [5]. The term “non-specific neck pain” refers to pain in the posterior region of the cervical spine, related to postural or mechanical causes without any specific origin such as trauma, whiplash-related disorders, sports-related injuries, and poor work ergonomics, and usually manifests without referred pain to the arms [6,7]. Patients who have neck pain for longer than three months are considered to have chronic non-specific neck pain [8]. Despite the widespread use of various treatments, relapses of neck pain are common, especially in chronic cases. This is largely due to the increasingly demanding nature of the modern lifestyle, such as the frequent usage of smartphones, laptops, and computers, which exposes the cervical region to various unnatural stresses [9]. With the increased incidence rate of neck pain, clinicians may inappropriately adopt a more complex approach while observing the surrounding structures, undertaking objective assessment and imaging [10]. As restoring neck movement is essential for the management of neck pain, it becomes even more difficult for patients to follow the prescribed exercise plan due to increased tenderness, as well as due to fear-avoidance and reluctance [11,12]. Therefore, in this context, the concept of biomechanical coupling can be particularly useful as it provides an indirect approach to achieving the target motion by using a separate motion segment (jaw movement). This motion may have the potential to be integrated into patients’ daily routines as a neck pain management strategy.

A similar mechanism exists between the upper cervical and temporomandibular joints, where opening and closing the jaw is linked to neck extension and flexion, respectively. This movement is accomplished by activating both the jaw and the neck muscles at the same time. This concept has led to the study of underlying physiological linkages between mandibular and cervical spine movements [13,14]. The fifth cranial nerve (CNV) innervates the orofacial tissues through primary afferent nociceptive and proprioceptive fibers [15]. Another author has suggested that the CNV receives sensory input from the various areas of the head, neck, and orofacial tissues, such as the head anterior portion, the greater occipital nerve, and branches of the upper cervical roots in the dorsum areas of the cervical spine [16]. It is important to understand that nociceptive or pain information from the CNV cervical stimulates the neurons in the area of the trigeminal nucleus caudalis, which expand to the area of the C2 spinal segment and lateral cervical nucleus in the posterior–lateral part of the cervical spine [16]. These authors further suggested that these neurons receive more than one type of afferent input and that there is an overlap between the CNV and cervical spine regions, called the convergence mechanism. Based on this mechanism, the CNV initiates symptoms in the area of the CNV and the cervical spine. Similarly, the cervical spine facilitates symptoms in the area of the cervical spine and CNV [16]. This close proximity of the trigeminal nerve and cervical spinal segments suggests that afferent activity from the jaw orofacial area has a substantial input to the head–neck motor control mechanism.

In the clinical context, the cervical spine and temporomandibular joint (TMJ) are interrelated, and the presence of dysfunction in one of the two areas influences mutual symptomatology; dysfunction in one region may result in adverse symptoms in the other region. The efficacy of physiotherapy treatments administered to the two regions revealed a marked improvement in temporomandibular and cervical pain by using medications and multiple types of physiotherapy techniques [17]. A previous study explored the frequency of cervical spine dysfunctions in people with temporomandibular disorders (TMDs). The results revealed an increased limitation of cervical flexion and extension, hypomobility at the facets of the joints and suboccipital area, and increased muscle sensitivity in the cervical, dorsal, and shoulder regions [18]. In a prolonged cervical flexion posture that is associated with stress, the mandibular condyle presses back against the meniscal tissue, causing pain, inflammation, and, eventually, meniscal tissue degeneration [19].

Some studies investigating neck pain have incorporated mandibular exercises into their treatment regimen. A study investigated the effect of adding TMJ treatments to routine physiotherapy in patients with non-specific chronic neck pain. The control group underwent soft tissue release, muscle energy techniques, stretches, endurance exercises, strengthening, and range of motion for the neck region. The intervention group received muscle energy techniques, soft tissue release, tongue movements, and stretches for the TMJ region, along with a routine physiotherapy regimen [20]. Another study involving patients with persistent craniocervical pain was divided into three groups: the exercise therapy (ET) group underwent jaw movement exercise (JME), the exercise therapy–psychological intervention (ET-PI) group received both JME and PI, and the control group received just pharmaceutical treatment. The authors came to the conclusion that jaw exercise alone is not as effective as jaw exercise with a psychological intervention to decrease parafunctional activities for improving craniocervical pain in patients without obvious organic problems [21]. In these studies, the authors applied multiple techniques to the TMJ, and it appears that few studies have explored the use of jaw movements to treat neck region dysfunction, despite the close functional relationship between the jaw and the head and neck area. During the jaw-opening movement, the neck extensor muscles become activated, and during the mouth-closing movement, the neck flexor muscles become activated [22]. Jaw and neck movements are innate in nature. This coordination between the jaw and neck muscles has been observed during fetal yawning [23]. Taking these factors together, it is well established that during jaw opening, the neck extensor muscles are employed and the jaw and neck flexor muscles are activated. Since there is a close functional association between the jaw and the upper cervical region, it is assumed that the jaw’s sensorimotor system could be useful in the treatment and management of neck pain. As a result, the goal of this study was to evaluate the effects of integrating jaw opening and closing movements along with active neck exercises versus active neck exercises alone in the management of chronic non-specific neck pain and to examine the effects of these treatments on neck pain, disability, endurance, and neck proprioception.

## 2. Materials and Methods

### 2.1. Study Design

This study was a randomized controlled trial with a double-blinded, two-armed, parallel design, with 40 participants per group.

### 2.2. Study Setting

This study was conducted at the Sindh Institute of Physical Medicine and Rehabilitation, Karachi, Pakistan.

### 2.3. Study Population

This study included patients with chronic non-specific neck pain.

### 2.4. Sample Size Calculation

There were no closely related previously published studies that would be suitable for sample size calculation on the same patients using jaw movement. Therefore, the power of the test was calculated to justify the sample size of 80 participants (40 per group) using PASS version 2021 software [24]. Based on a repeated-measures two-way analysis of variance (with two levels between and two levels within the group) with a 95% confidence interval, a numerical pain rating scale (NPRS) within-effect size of 1.062, and a between-effect size of 1.363, the result was found to be greater than 99%. The same power of the test was found for other outcome measures using the same confidence interval, with a within-effect size and between-effect size for other outcomes: neck disability index (NDI), 2.398 and 5.934; neck flexion endurance (NFE), 1.574 and 3.027; neck extension endurance (NEE), 2.683 and 6.303; neck flexion proprioception error (NFPE), 1.027 and 1.575; neck extension proprioception error (NEPE), 0.812 and 0.451; neck right rotation proprioception error (NRRPE), 0.893 and 1.72; and neck left rotation proprioception error (NLRPE), 1.045 and 1.701.

### 2.5. Study Duration

The duration of this study was from November 2018 to October 2022.

### 2.6. Data Collection Procedure

All subjects were enlisted and diagnosed with chronic non-specific neck pain by consultants possessing a fellowship degree and five years of experience in the fields of neurology, neurosurgery, orthopedics, and rheumatology. These consultants referred patients on the basis of the following inclusion and exclusion criteria. The inclusion criteria included both male and female participants with ages between 18 and 50 years, neck pain for more than 3 months, and no comorbidities. The exclusion criteria in this study included patients with a history of a specific cause of neck pain, TMJ dysfunction, whiplash-associated disorders, cervical spondylosis, rheumatoid arthritis, instability of the spine, facial injury or dental infection, neck or spinal segment fracture, spinal tumors, unexplained headache, post-cervical spine surgery, cervical spine stenosis, cervical disc bulge or disc herniation, radiculopathy of the cervical spine, cognitive impairment, neurological conditions (MS/PD/CVA/MND), the application of injection therapy in the cervical spine, and red flags (double vision, dysarthria, dysphasia, drop attacks, dizziness, and gait disturbance).

Furthermore, the physiatrist reconfirmed the diagnosis of chronic non-specific neck pain in order to make a referral to the physiotherapy department. All participants received detailed information about the research study and each participant signed a written consent form before being directed to the physiotherapy department.

These criteria played a role in refining and narrowing down the diagnostic standards for chronic non-specific neck pain. A total of 80 participants, with 40 participants in each group, were enrolled in the study. Group A patients were assigned jaw movements (mouth opening and closing) with active neck exercises, while Group B patients were assigned only active neck exercises. Isometric strengthening neck exercises with a home exercise plan were given to both groups.

### 2.7. Randomization and Recruitment

**Figure 1:**
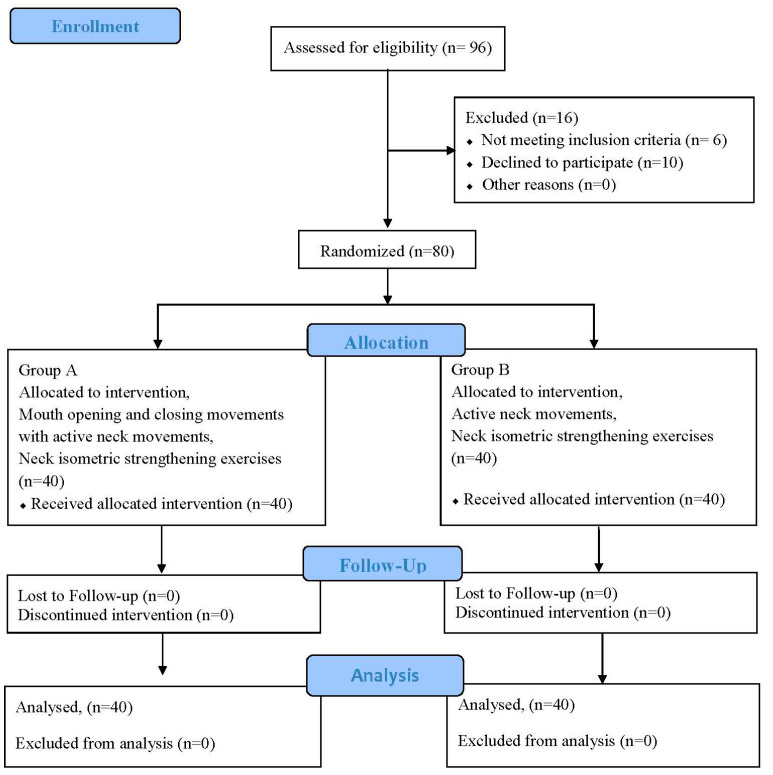
CONSORT diagram.

The sample was drawn randomly by a non-probability purposive sampling technique with simple randomization, using a computer-generated Microsoft Excel sheet (Microsoft Office 365, Microsoft, Redmond, WA, USA). In total, 96 patients were referred by various consultants with a confirmed diagnosis of chronic non-specific neck pain; however, 16 patients were excluded from the study because they had not met the eligibility criteria or declined to participate. Therefore, a total of 80 patients were recruited and divided into two groups (Group A and Group B) (Figure 1).

### 2.8. Blinding

In this study, a senior physiotherapist evaluated the participants using specified outcome measures, attending as an outcome assessor at the baseline and final sessions. The outcome assessor was blinded to the respective treatments assigned to the study participants. Participants were also not made aware of which group they were assigned to, and they had an equal chance of being allocated to either group A or B, as mentioned in the consent form. It was further stated in the form that all participants would receive exercise therapy for neck pain management without disclosing any description of treatment per group, as part of the blinding process. Participant blinding to the treatment was further optimized by ensuring that both groups received assessment and treatment sessions on separate days to avoid participant interaction and the disclosure of their respective treatment plans to each other. Additionally, participants were requested not to discuss their therapy with the outcome assessor.

### 2.9. Interventions

#### 2.9.1. Experimental Group

Each patient was seated on a comfortable chair with a back support, their feet on the floor, and with no head support. The patient was then asked to actively perform self-paced maximal jaw opening and closing movements in such a way that there was a coordinated neck extension during jaw opening and neck flexion during jaw closing. All patients performed 3 sets of 15 repetitions, with 2 min of rest in between each set of the exercises. All subjects received a total of 18 sessions over a 6-week period, including the initial and final assessment sessions:

- Initial assessment with outcome measures recorded at baseline (week 1), in a session of 60 min;
- 2 treatment sessions (week 1), 40 min duration;
- 3 treatment sessions per week (week 2–5), 40 min duration;
- Last 2 treatment sessions (week 6), 40 min duration;
- Final assessment with outcome measures recorded at the final session (week 6), 60 min duration.

#### 2.9.2. Control Group

Each patient was seated on a comfortable chair with back support, their feet on the floor, and with no head support. The patient was then asked to actively perform self-paced neck extension and flexion movements without incorporating jaw movement. All patients performed 3 sets of 15 repetitions, with 2 min of rest in between each set of the exercises. All subjects received a total of 18 sessions over a 6-week period, including initial and final assessment sessions.

The initial assessment, with outcome measures recorded at baseline session (week 1), lasted for 60 min:

- 2 treatment sessions (week 1), 40 min duration;
- 3 treatment sessions per week (week—2–5), 40 min duration;
- Last 2 treatment sessions (week 6), 40 min duration;
- Final assessment with outcome measures recorded at the final session (week 6), 60 min duration.

#### 2.9.3. Supportive Treatments

Both the experimental and control groups received isometric strengthening exercises with manual resistance from the therapist, performing cervical flexion, extension, and rotation exercises in the above-mentioned position. There were 3 sets (15 repetitions) with 2 min of rest in between. Self-resisted strengthening exercises for cervical extensors, flexors, and lateral flexors (15 reps with a 6-second hold, 3 times per day) were provided as a home exercise plan. In addition, each patient received a postural advice sheet and a home diary to keep track of their exercise program.

### 2.10. Outcome Measures

The outcome measures used in this study were the NPRS, NDI, NFE, NEE, and the NPE: NFPE, NEPE, NRRPE, and NLRPE tests. The following outcome measures were taken at the baseline (week 1) before treatment and at the final session after the last treatment session (week 6).

#### 2.10.1. Numeric Pain Rating Scale

Patients marked the pain intensity in the NPRS diagram, ranging from 0 to 10 on an ordinal scale; one of the most frequently used outcome measures for determining pain severity, as detailed in a previous study [25]. The NPRS has shown high-to-moderate quality of evidence, with moderate to strong test-retest reliability [26,27].

#### 2.10.2. Neck Disability Scale

In this study, a validated Urdu version of the NDI was used, based on which the patients marked those levels in each component of disability that corresponded to their perceived levels of disability, with total scores calculated for the initial and final sessions [28]. This was used in addition to its original English version, as needed.

The NDI questionnaire consists of 10 different domains of questions, which are used to assess neck disability caused by neck dysfunction. Pain, personal care (washing and dressing), lifting, reading, headaches, concentration, work, driving, sleeping, and recreational tasks are among these 10 domains. Each question item is scored from 0 to 5. A score of ‘0’ indicates no pain or limitation, while a score of ‘5’ indicates the most pain or limitation in a task.

The maximum possible score is 50. The scoring breaks for analysis are as follows: no disability (0–4), mild (5–14), moderate (15–24), severe (25–34), and total disability (above 34) [29]. The NDI and standard pain and disability scores were linked favorably. Without a floor or ceiling effect, the NDI showed strong test-retest reliability and internal consistency [30].

#### 2.10.3. Neck Muscle Endurance Tests

A previous study revealed moderate reliability (ICC = 0.67) for the neck flexor endurance test, while another study revealed a good reliability value for the neck extensor endurance test, where Kappa = 0.80 [31,32].

##### Neck Flexor Endurance Test

The patient was asked to tuck their chin in, isometrically, and then elevate the occiput 1 in from the couch surface, while lying supine on a couch with both knees flexed and feet together [31]. The two natural horizontal creases formed over the lateral aspect of the patient’s neck were marked in this position. The patient was then instructed to keep his or her head in this elevated position for as long as possible without assistance. The test was terminated when the patient was no longer able to keep their head in the stated position and the movement of the two skin creases away from each other. A stopwatch was used to record the duration of this isometric neck flexion. This phase was repeated twice, with a five-minute break between each repetition, and the averages of 2 measurements were used for data analysis.

##### Neck Extensor Endurance Test

In the prone position, the patient was instructed to extend his or her neck beyond the edge of the couch while keeping the upper chest and cervico-thoracic region stabilized against the couch. The patient then tucked in his or her chin while in this neutral position. The test was terminated when the patient was no longer able to maintain the chin tuck-in position or there was an increase in cervical lordosis. A stopwatch was used to record their time in seconds [32]. This phase was repeated twice, with a 5-minute break between each set, and the averages of 2 measurements were used for data analysis.

#### 2.10.4. Neck Proprioceptive Error Test

The proprioception error was assessed through range of motion measurement using the Cervical Range of Motion Device^®^, ‘Performance Attainment Associates, St. Paul, MN, USA’, which is a reliable way to measure range of motion. Three inclinometers were included in the CROM device, two of which were fixed or nonadjustable. The sagittal plane’s flexion and extension, as well as the frontal plane’s lateral flexion, were measured using the gravity-dependent fixed nonadjustable inclinometer. The third movable inclinometer monitored the neck rotation in the transverse plane and was operated by a compass. The magnetic collar used in this setup had to be fastened around the participant’s upper trunk [33]. The proprioception error was assessed through a range of motion measurement using the Cervical Range of Motion Device^®^, which demonstrated a good level of reliability and validity [34,35].

The patient was seated upright, wearing opaque goggles with their eyes closed to block any visual input, and was instructed to precisely reposition their head back into a starting position. The Cervical Range of Motion Device^®^ was placed on the patient’s head and a neutral reference point or NRP, i.e., starting position, was recorded for each plane of the cervical spine [36].

Taking the above protocol, the patient’s cervical range of motion was then measured in the following order:

Right-sided rotation of cervical spine: turn to look over your right shoulder
Left-sided rotation of cervical spine: turn to look over your left shoulder
Flexion of cervical spine: bending the head forward toward the chest
Extension of cervical spine: bringing the head backward

Each of the above movements was performed in 3 sets with a 60-s interval in between each set of movements. Each movement was completed in a half-cycle, with the head returned to the NRP or starting position [37]. Patients were asked to move their heads until they believed they had achieved the NRP or starting position. With reference to the NRP, the difference in movement magnitude during each attempt was calculated as under-shoot or over-shoot and was considered a ‘proprioceptive error’. An average ‘proprioceptive error’ value for each cervical movement was calculated, regardless of under-shoot (−ve) or over-shoot (+ve) values.

### 2.11. Statistical Analysis

IBM SPSS version 27.0 (IBM Inc., Armonk, NY, USA) was used to analyze the data. The mean and standard deviations for age, NPRS, NDI, NFE, NEE, NFPE, NEPE, NRRPE, and NLRPE were calculated. Gender was reported in terms of frequency and percentage. The baseline demographic characteristics were compared using a two-sample independent *t*-test, and the chi-square test was used to see if there was an association between groups with gender and pain duration. After establishing the normality of the data distribution, a repeated-measures two-way ANOVA was used to determine the mean difference within the groups for all outcome measures. The least significant difference (LSD) test was used for pairwise comparisons. Significant was defined as a *p*-value of 0.05 or less.

## 3. Results

Out of 80 participants, there were 40 (50%) males and 40 (50%) females with a mean age of 35.6 ± 8.5 years in the experimental group; in the control group, it was 36.1 ± 8.7 years. All 80 participants completed their treatment sessions for six weeks and no harmful effects were observed. Participants with pain duration ranging from 3 to 6 months, 6 to 9 months, 9 to 12 months, and > 15 months were equal to or greater than 50% in the control group, except for patients with pain duration ranging from 12 to 15 months, who were equal in both groups. In terms of age, gender, and pain duration, there was no significant difference (*p*-value > 0.05) between the two groups (Table 1).

NPRS and NDI showed a significant difference in the CROM measurements but mean improvements post-intervention for CROM were better in the experimental group compared to the control group. NPRS (F1,78 = 27.93, MSE = 1.128, *p* < 0.001, and $\eta_{p}^{2}$ = 0.264) and NDI (F1,78 = 61.048, MSE = 5.750, *p* < 0.001, and $\eta_{p}^{2}$ = 0.439). Post-intervention, for both NPRS and NDI, we found a clinically significant mean difference between groups (Cohen’s d = 1.262 and Cohen’s d = 1.422, respectively) (Table 2).

In the experimental group, a significant group-by-time interaction was seen in endurance NFE (F1,78 = 40.599, MSE = 2.476, *p* < 0.001, and η_p^2 = 0.342,) and for NEE (F1,78 = 67.549, MSE = 7.196, *p* < 0.001, and η_p^2 = 0.464). At post-intervention for both NFE and NEE, we found a clinically significant mean difference between groups (Cohen’s d = −1.056 and Cohen’s d = −1.174, respectively) (Table 3).

The mean comparison of proprioception error was reported, which showed a considerable improvement in the experimental group than the control group in NFPE (F1,78 = 4.868, MSE = 1.054, *p* = 0.030, and η_p^2 = 0.059), for NEPE (F1,78 = 15.370, MSE = 0.660, *p* < 0.001, and η_p^2 = 0.165), for NRRPE (F1,78 = 7.514, MSE = 0.797, *p* = 0.008, and η_p^2 = 0.088), for NLRPE (F1,78 = 4.703, MSE = 1.091, *p* = 0.033, and η_p^2 = 0.057). At post-intervention for NEPE, NRRPE, and NLRPE, we found a clinically significant mean difference between groups (Cohen’s d = 0.740, Cohen’s d = 0.627, and Cohen’s d = 0.615, respectively) (Table 4).

## 4. Discussion

In the present study, patients performing active jaw movements as an adjunct to active neck exercises reported significant improvements in terms of pain intensity, neck disability, neck muscle endurance, and neck proprioception. The improvement evinced in these outcomes was significantly greater when the exercises were coupled with jaw opening and closing movements than when the exercises were performed in isolation. The present study generated evidence to suggest that jaw opening and closing movements are invaluable in improving chronic non-specific neck pain and no adverse effect was observed in this study.

Similar results were found in a study conducted in Japan, where jaw movements in conjunction with psychological intervention were found to decrease craniometrical pain [21]. However, despite having the advantage of being safe to administer, exercise therapy may not be sufficient to manage the condition in isolation without considering the biomechanically relevant structures, such as the jaw. This study is unique in adopting a more holistic approach, in which the biomechanical link between the jaw and neck was exploited and utilized as a treatment strategy. This interconnection is substantiated by the fact that jaw opening and closing movements are always accompanied by head–neck extension and flexion, respectively. However, simultaneous activation of jaw and neck muscles is required to achieve functional movements in this region [14]. A high correlation between TMJ dysfunction and neck disability further corroborates this approach [38]. The clinical evidence of any trauma-related cause-and-effect association between whiplash and TMDs was compiled by the authors of [39,40]. In terms of reducing pain, raising pressure pain thresholds, and improving the cervical range of motion, physiotherapy based on aerobic exercise, in conjunction with temporomandibular joint exercises such as the mouth-opening movement and a movement with the mouth opening in alignment and sideways proved to be more successful than aerobic exercise alone in the physiotherapy treatment program [41].

In the present study, better outcomes were observed in the group performing jaw opening and closing movements with active neck exercises (flexion and extension); this could also be explained by the neuroanatomical connections between the two regions [16]. It has also been suggested that jaw and neck muscle actions are coordinated by simultaneous neural commands to both the jaw and the neck motor systems. The neural circuitry for this integrated jaw and neck function is innate and remains unchanged throughout life [42]. The present study was also unique in employing multiple subjective and objective outcome measures to evaluate the efficacy of the interventions comprehensively. Many authors have used NPRS and NDI in their studies as subjective outcome measures [43,44,45].

Among the subjective tools used, the NPRS and NDI recorded 73% and 57% improvements in self-reported pain and disability, respectively, in the experimental group, and these findings demonstrated a clinically significant mean difference between groups at the post-intervention for both the NPRS and the NDI. Another study by Mariam Tariq et al. found NPRS and NDI scores to be 14.9% and 12.4%, respectively, after two weeks of isometric exercises in patients with mechanical neck pain. They also found the ‘active range of motion exercises’ to be less effective than isometric exercises in decreasing pain and disability in such patients [46]. Conversely, Deen et al. found that the combination of static stretching with isometric exercise yielded a 60% improvement in NPRS scores and a 75% improvement in NDI scores in patients with chronic neck pain [47].

The current study also reported substantial improvement in the endurance of neck flexors (152%) and neck extensors (83%) in the experimental group. In the context of this finding, our study revealed that following the intervention, a clinically statistically significant mean difference was seen between the groups for both NFE and NEE. A previous study revealed significant improvements in cervical muscle endurance, pain, and disability with three weeks of cervical endurance training than with isometric exercises for mechanical neck pain [48]. However, another study reported that a six-week stretching and strengthening program for cervical muscles improved endurance and range of motion and decreased cervical pain [49].

This may indicate that better outcomes of isometric training will become apparent in relatively longer follow-ups. The present study also reported improvements in both groups in terms of proprioception errors in the sagittal and transverse planes, including cervical flexion, extension, right rotation, and left rotation, with significantly higher scores in the experimental group. A clinically statistically significant mean difference was observed between the groups for NFPE, NEPE, NRRPE, and NLRPE following the treatment sessions. While this finding attests to the efficacy of the accompanying jaw movements, it also advocates the use of a multi-planner approach for isometric strengthening exercises, a method employed in the present study. This is in line with the findings reported by Shoukat et al., who stated that multiple-angle neck isometrics are more effective in improving neck pain and disability than isometric neck exercises performed with a neutral spine [50].

These results were made possible by the meticulous design of this research study, where various factors were taken into consideration. While an increased number of sessions ensured the effective administration of the treatment to each group, the use of validated objective outcome tools in addition to subjective tools allowed for a comprehensive study of neck pain. However, the present study is by no means devoid of limitations, and the authors recommend drawing upon a larger sample to allow stratifications according to the individual age groups and different durations of neck pain, i.e., acute, subacute, and chronic, to achieve further insights into the outcomes in the long term.

The outcomes of our study have highlighted the role of the jaw somatosensory system and its clinical implications regarding cervical pain. It has been observed in clinical practice that many patients have great difficulty in performing neck exercises smoothly, due to fear avoidance and increased tenderness, which also prevent the application of hands-on techniques such as manipulation, mobilization, soft tissue glides, etc. In this regard, jaw movements could prove to be a useful adjunct to any active exercise regimen for neck pain, as evidenced by the promising results observed in this study. This addition could also be instrumental in restoring normal cervical movement in the sagittal and transverse planes, which are commonly impaired in neck pain.

The main strengths of the study include the careful design of this research study, where several factors, including randomization, recruitment, blinding, etc., were kept under consideration, along with consort guidelines in relation to controlled confounding factors. The same investigator administered the intervention to both groups. The absence of more long-term follow-up, where the outcomes are studied after several months, is one of the limitations of the study. For future studies, it is recommended that outcomes are also studied 3–6 months after the conclusion of the treatment sessions.

Additionally, a larger sample should be recruited to allow stratifications according to the individual age groups and different durations of neck pain, i.e., acute, sub-acute, and chronic.

## 5. Conclusions

Integrating jaw opening and closing movements along with active neck exercises is more effective than active neck exercises alone for the treatment of patients with chronic non-specific neck pain. This study proposes a novel physiotherapy treatment approach for the management of chronic nonspecific neck pain and suggests that the synchronization of active neck exercises with jaw opening and closing movements is of great significance in restoring mobility, reducing neck pain and disability, improving neck muscles endurance and normalizing neck proprioception in such patients.

## Figures and Tables

**Table 1:** Demographic and clinical characteristics among groups.

Characteristics	Experimental N = 40 (%)	Control N = 40 (%)	*p*-Value
**Gender**			
Male	20 (50)	20 (50)	>0.99 ^c^
Female	20 (50)	20 (50)	
**Age (years),** mean ± SD	35.6 ± 8.5	36.1 ± 8.7	0.806 ^I^
**Pain duration (months)**			
3–6	3 (42.9)	4 (57.1)	0.909 ^c^
6–9	5 (45.5)	6 (54.5)	
9–12	7 (50.0)	7 (50.0)	
12–15	16 (57.1)	12 (42.9)	
>15	9 (45.0)	11 (55.0)	

^c^ Chi-square test, ^I^ independent *t*-test, SD: standard deviation.

**Table 2:** Mean comparison of the numeric pain rating scale and neck disability index within groups and between groups.

Outcome Measures	Experimental (N = 40)	Control (N = 40)	Cohen’s d	Control vs. Experimental ^MD^
**Neck Pain Rating Scale**				
Pre ^MS^	6.68 ± 0.83	6.78 ± 0.92	0.114	0.1 (0.611)
	(6.40, 6.95)	(6.50, 7.05)		
Post ^MS^	1.80 ± 1.49	3.68 ± 1.49	1.262	1.88 (<0.001)
	(1.33, 2.27)	(3.21, 4.14)		
Pre vs. Post ^MD^	4.88 (<0.001)	3.10 (<0.001)		-
Improvement (%)	73	46		-
**Neck Disability Index**				
Pre ^MS^	24.35 ± 4.60	25.13 ± 0.920	0.235	0.78 (0.426)
	(22.99, 25.71)	(23.76, 26.49)		
Post ^MS^	10.70 ± 4.58	17.40 ± 4.840	1.422	6.7 **(<0.001)**
	(9.22, 12.18)	(15.92, 18.88)		
Post vs. Pre ^MD^	−13.65 (<0.001)	−7.73 (<0.001)		-
Improvement (%)	57	32		-

^MS^ Values represented as mean ± standard deviation (95% C.I). ^MD^ Values represented as the mean difference (*p*-value).

**Table 3:** Mean comparison of neck flexion endurance and neck extension endurance within groups and between groups.

Outcome Measures	Experimental (N = 40)	Control (N = 40)	Cohen’s d	Control vs. Experimental ^MD^
**Neck Flexion Endurance**				
Pre ^MS^	5.06 ± 1.37	5.1 ± 1.64	0.026	0.04 (0.90)
	(4.59, 5.54)	(4.63, 5.59)		
Post ^MS^	12.4 ± 3.09	9.29 ± 2.79	−1.056	−3.13 **(<0.001)**
	(11.45, 13.38)	(8.32, 10.25)		
Pre vs. Post ^MD^	−7.35 (<0.001)	−4.18 (<0.001)		-
Improvement (%)	152	88		-
**Neck Extension Endurance**				
Pre ^MS^	13.44 ± 2.66	13.33 ± 3.72	−0.034	−0.12 (0.872)
	(12.43, 14.47)	(12.32, 14.35)		
Post ^MS^	24.78 ± 7.10	17.69 ± 4.75	−1.174	−7.09 **(<0.001)**
	(22.88, 26.68)	(15.79, 19.59)		
Pre vs. Post ^MD^	11.33 (<0.001)	4.36 (<0.001)		-
Improvement (%)	83	34		-

^MS^ Values represented as mean ± standard deviation (95% C.I). ^MD^ Values represented as the mean difference (*p*-value).

**Table 4:** Mean comparison of proprioception error within groups and between groups.

Outcome Measures	Experimental (N = 40)	Control (N = 40)	Cohen’s d	Control vs. Experimental ^MD^
**Neck Flexion Proprioception Error**				
Pre ^MS^	4.63 ± 1.54	4.38 ± 1.50	−0.164	−0.25 (0.464)
	(4.15, 5.11)	(3.9, 4.86)		
Post ^MS^	1.80 ± 1.08	2.26 ± 1.14	0.414	0.47 (0.063)
	(1.45, 2.14)	(1.91, 2.61)		
Post vs. Pre ^MD^	−2.83 (<0.001)	−2.12 (<0.001)		-
Improvement (%)	58	48		-
**Neck Extension Proprioception Error**				
Pre ^MS^	4.80 ± 1.26	4.55 ± 1.43	−0.186	−0.25 (0.410)
	(4.38, 5.23)	(4.13, 4.98)		
Post ^MS^	1.62 ± 0.72	2.379 ± 1.26	0.740	0.76 **(0.001)**
	(1.3, 1.94)	(2.06, 2.7)		
Post vs. Pre ^MD^	−3.18 (<0.001)	−2.18 (<0.001)		-
Improvement (%)	65	48		-
**Neck Right Rotation Proprioception Error**				
Pre ^MS^	4.49 ± 1.52	4.48 ± 1.50	−0.007	0.01 (0.981)
	(4.01, 4.96)	(4.01, 4.96)		
Post ^MS^	1.51 ± 0.93	2.27 ± 1.44	0.627	0.72 **(0.006)**
	(1.12, 1.89)	(1.89, 2.65)		
Post vs. Pre ^MD^	−2.99 (<0.001)	−2.21 (<0.001)		-
Improvement (%)	65	51		-
**Neck Left Rotation Proprioception Error**				
Pre ^MS^	4.51 ± 1.85	4.44 ±1.50	−0.042	−0.06 (0.877)
	(3.98, 5.04)	(3.92, 4.98)		
Post ^MS^	1.46 ± 0.75	2.12 ± 1.32	0.615	0.66 **(0.008)**
	(1.13, 1.8)	(1.78, 2.46)		
Post vs. Pre ^MD^	−3.04 (<0.001)	−2.33 (<0.001)		-
Improvement (%)	62	52		-

^MS^ Values represented as mean ± standard deviation (95% C.I), ^MD^ Values represented as mean difference (*p*-value).

## Data Availability

The data presented in this study are available on request from the corresponding author. The data are not publicly available due to privacy issues.
